# Phytochemical Analysis, Antimicrobial, and Antioxidant Activities of North Macedonia *Achillea setacea* Essential Oil

**DOI:** 10.3390/antiox15070820

**Published:** 2026-06-29

**Authors:** Antonella Porrello, Alessia Sordillo, Giusy Castagliuolo, Dario Antonini, Gianfranco Fontana, Natale Badalamenti, Mario Varcamonti, Maurizio Bruno, Vincenzo Ilardi, Anna Zanfardino

**Affiliations:** 1 Department of Biological, Chemical and Pharmaceutical Sciences and Technologies (STEBICEF), University of Palermo, Viale Delle Scienze, Ed. 17 Stanislao Cannizzaro, 90128 Palermo, Italy; antonella.porrello@unipa.it (A.P.); gianfranco.fontana@unipa.it (G.F.); maurizio.bruno@unipa.it (M.B.); vincenzo.ilardi@unipa.it (V.I.); 2 Department of Biology, University of Naples Federico II, 80126 Naples, Italy; alessiasordillo2015@gmail.com (A.S.); giusy.castagliuolo@unina.it (G.C.); dario.antonini@unina.it (D.A.); varcamon@unina.it (M.V.); anna.zanfardino@unina.it (A.Z.)

**Keywords:** *Achillea setacea*, Asteracee, Caco-2 cells, camphor, eucalyptol, 4-terpineol

## Abstract

The complex genus *Achillea* L. comprises more than 140 species distributed widely throughout the Northern Hemisphere. Several species are widely used in traditional medicine for their therapeutic properties, yet few studies have correlated their biological properties with the plant’s phytochemical composition. Among these, *Achillea setacea* Waldst. & Kit. is a perennial species traditionally used to treat digestive and inflammatory disorders. In this study, the essential oil of *A. setacea*, collected wild in North Macedonia, was analyzed spectrometrically and spectroscopically by GC-MS and NMR, respectively. A total of nineteen compounds were identified, with camphor (31.3%), 4-terpineol (11.3%), and eucalyptol (10.6%) being the main constituents. Furthermore, the biological activities of pure oil were evaluated, showing notable antioxidant properties, as well as antimicrobial effects against a panel of clinically relevant microorganisms, including Gram-positive and Gram-negative bacteria. Furthermore, its impact on human intestinal epithelial (Caco-2) cells was assessed, highlighting its potential relevance for gastrointestinal applications, in agreement with the traditional use of *Achillea* species for digestive disorders.

## 1. Introduction

The genus *Achillea* L. includes between 141 and 153 species, depending on the different plants database [1,2]. It is native to the temperate and subtropical regions of the Northern Hemisphere, and it is typical of Mediterranean climate regions [1,2]. The name *Achillea* arises from one of the heroes of the Iliad, Achilles, who is said to have used these plants to treat soldiers by stopping bleeding and healing wounds; in fact, accordingly, in folk medicine, *Achillea* is regarded as one of the most well-known hemostatic plants [3]. Several species of this genus have been largely utilized in the popular ethno-medicine of several countries. Their uses include dyspeptic disorders, gynecological pain, gastritis, colds, fever, stomach aches, ulcers, and psychosomatic cramp pains, and the medicinal properties of this genus have been reviewed [4,5].

**Figure 1:**
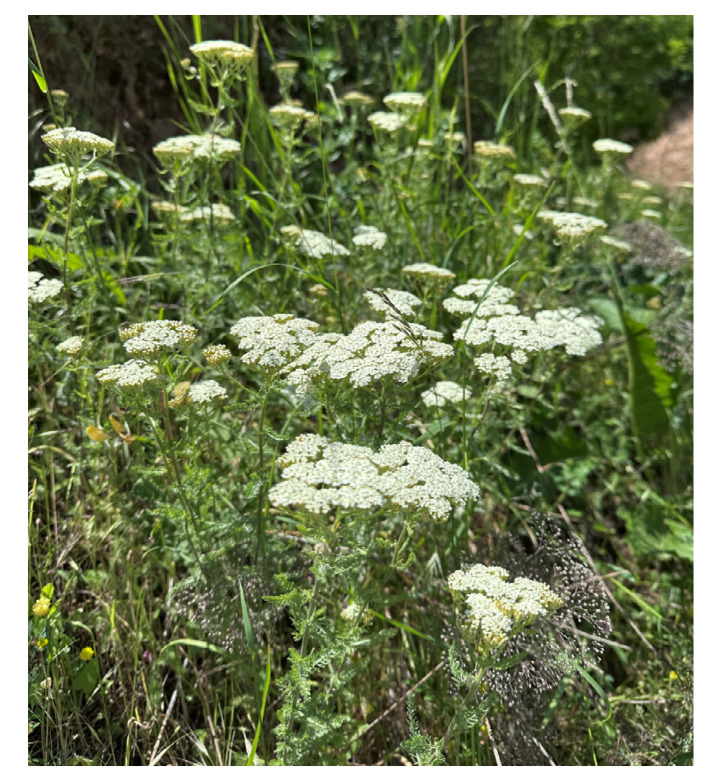
*Achillea setacea* Waldst. & Kit. Photo by Dr. Natale Badalamenti.

*Achillea setacea* Waldst. & Kit. (Figure 1), belonging to the *Achillea millefolium* group, is a perennial and grows primarily in the temperate biome, whose native range is from Europe to Korea and the Arabian Peninsula [2]. The stems are 15–30 cm long, erect or ascending, simple, with 12–20 internodes. The middle leaves are cauline (3 × 0–5 cm), narrowly lanceolate in outline, 3-pinnatisect, and pubescent to sericeous. The involucre is c. 3 × 1.5–2.5 mm with bracts more or less evenly pubescent. The corymbs usually have many capitula. The ligules are white. It grows mainly in dry places [6].

*Achillea setacea* has been utilized in Turkey as a folk medicine for the treatment of gynecological diseases, skin diseases, digestive system disorders, and tuberculosis [7], and furthermore, its dried flowers, known among Turkish people as “ayvadana”, “ayvadani”, and “mayasılotu”, are used internally as an infusion for hemorrhoids, as an anti-diarrheal and wound-healing agent, and also against abdominal pain [8]. Its specific properties are often associated with those of the *Achillea millefolium* complex. These species have been largely utilized, both internally and externally, for treating wounds, stopping bleeding, as well as for treating colds, fevers, kidney diseases, and menstrual pain [9,10]. The herb mixed with *Sambucus nigra* L. flowers and *Mentha* x *piperita* L. has been utilized for treating colds and influenza [11]. Furthermore, its antiseptic, antispasmodic, vasodilator, digestive, diaphoretic, emmenagogue, odontalgic, and vulnerary properties have been reported [9,10]. The fresh leaf applied directly on an aching tooth is reported to relieve pain [11], and the herb is also used as food; leaves are eaten raw or cooked and utilized as a hop substitute for flavoring and as a preservative for beer [12,13,14].

Previous phytochemical investigation on the non-volatile metabolites of *A. setacea* showed the occurrence of sesquiterpenoids [15,16], flavonoids, and phenolic compounds [17,18,19]. As for the essential oil compositions, several studies [20,21,22,23,24,25,26,27,28] have been carried out on the essential oil of *A. setacea*, but none of them concern the North Macedonian accession. These results will be discussed later. In this context, the present study aims to investigate the biological properties of the essential oil of *A. setacea* (**As**), focusing on its antioxidant, antimicrobial, and cell-related activities. Antimicrobial testing was performed against a panel of microorganisms selected for their clinical relevance and involvement in infections associated with skin, gastrointestinal, and gynecological disorders. In particular, *Staphylococcus aureus*, *Staphylococcus epidermidis* and *Pseudomonas aeruginosa* are among the most common pathogens implicated in skin and soft tissue infections and wound colonization [29,30]. In addition, *Escherichia coli* and *Bacillus cereus* are well-known causative agents of gastrointestinal infections and foodborne diseases [31]. Finally, *Candida albicans* is an opportunistic fungal pathogen responsible for mucosal and gynecological infections, including vaginal candidiasis [32]. The choice of these pathogens is consistent with the traditional uses of *Achillea* species as antiseptic, wound-healing, and anti-infective remedies. In addition, the effects on Caco-2 human intestinal cells were evaluated to assess the potential cytotoxicity and relevance of the essential oil for gastrointestinal applications, considering the key role of intestinal cells in maintaining gut barrier integrity and intestinal homeostasis. Overall, this study provides new insights into the pharmacological potential of *A. setacea* essential oil from a previously unexplored geographical origin.

## 2. Materials and Methods

### 2.1. Plant Material

Aerial parts (flowers, leaves, and stems) of *Achillea setacea* Waldst. & Kit. were collected near Tetovo on Mt. Popova Shapka, North Macedonia, 1550 m a.s.l. (42°01′10” N, 20°54′06” E), on 27 March 2025. One of the samples, identified by Prof. Vincenzo Ilardi, has been stored in the Herbarium Mediterraneum Panormitanum (PAL) (Voucher N.109801) of the Botanical Garden of the University of Palermo, Italy.

### 2.2. Isolation of Essential Oil

Fresh aerial parts (flowers, leaves, and stems, totalling 900 g) were subjected to hydrodistillation for 3 h, according to the standard procedure described in the European Pharmacopoeia [33]. The sample, dehydrated with sodium sulfate (Na_2_SO_4_), was stored at 4 °C in a refrigerator in a vial equilibrated under a N_2_ atmosphere. Samples yielded 0.12% of essential oil.

### 2.3. GC-MS Analysis

Analysis of essential oil was carried out according to the procedure reported by Vaglica et al. [34]. Linear retention indices (LRIs) were calculated using a mixture of pure *n*-alkanes (C_8_–C_30_), and all the peaks’ compounds were identified by comparison with MS and by comparison of their relative retention indices with the WILEY275, NIST 17, ADAMS, and FFNSC2 libraries.

### 2.4. NMR Experiments

^1^H- and ^13^C-NMR spectra were recorded as reported in Castagliuolo et al. 2026 [35]. Deuterated chloroform (CDCl_3_), *n*-alkanes (C_8_–C_30_), eucalyptol, 4-terpineol, camphor, and pentane were purchased from Sigma-Aldrich (Milan, Italy).

### 2.5. Bacteria Selection

The microbiological effect was evaluated using the following bacterial strains: Gram-negative: *Escherichia coli* DH5α and *Pseudomonas aeruginosa* PAOI ATCC15692; Gram-positive: *Staphylococcus aureus* ATCC6538P, *Staphylococcus epidermidis* ATCC12228, and *Bacillus cereus* ATCC10987; the fungus *Candida albicans* ATCC14053, and the mycobacterium *Mycobacterium smegmatis* MC^2^155.

### 2.6. Minimum Inhibitory Concentration (MIC)

The minimum inhibitory concentrations (MICs) of **As** (resuspended in DMSO, 50%) and its major constituents (camphor, eucalyptol, and 4-terpineol) against selected microbial strains were determined using the broth microdilution method, according to the guidelines of the Clinical and Laboratory Standards Institute (CLSI) [36].

Briefly, 5 µL of bacterial suspension of 5 × 10^5^ CFU/mL were inoculated into 96-well microplates containing Mueller–Hinton broth and appropriate volumes of **As** or the individual compounds to obtain the desired final concentrations (0–20 mg/mL).

The volume of each test sample was calculated according to the dilution equation C_1_V_1_ = C_2_V_2_, and the volume of broth was adjusted accordingly to obtain a final volume of 200 µL per well. The tested concentrations of the single constituents were selected to reflect their relative abundance in **As** (camphor 31.3%, eucalyptol 10.6%, and 4-terpineol 11.3%). After incubation at 37 °C overnight, bacterial growth was assessed by measuring the optical density at 600 nm (OD600) using a microplate reader. The MIC was defined as the lowest concentration of the tested compound that resulted in complete inhibition of visible microbial growth. The MIC values of positive controls were evaluated using colistin against *P. aeruginosa* and *C. albicans*, and ampicillin for the other strains. All experiments were performed in triplicate, and results are expressed as the mean of three independent experiments.

### 2.7. Bacterial Lysis Assay

Bacterial lysis activity was evaluated according to a previously described method, with minor modifications [35]. *E. coli* and *S. aureus* were used as representative Gram-negative and Gram-positive model strains, respectively. Overnight cultures were diluted to an optical density at 600 nm (OD600) of 0.1 and incubated at 37 °C under shaking conditions until reaching mid-log phase (OD600 ≈ 1.0). Cells were then harvested by centrifugation (5000× *g*, 5 min), washed, and resuspended in phosphate-buffered saline (PBS, pH 7.4) to a final OD600 of 0.5. Untreated cells were used as growth controls. Cells treated with DMSO (50% *v*/*v*) and SDS (1% *w*/*v*) were used as negative and positive lysis controls, respectively. Bacterial suspensions were exposed to **As** at concentrations corresponding to the previously determined MIC values for each strain. Samples were incubated at 37 °C under continuous agitation, and OD600 was recorded at regular time intervals over a 4 h period using a microplate reader. Bacterial lysis was expressed as a percentage according to the following equation:


Bacterial lysis (%) = 100 × [1 −(OD_treated_/OD_(control)_)]

All experiments were performed in triplicate.

### 2.8. Fluorescence Microscopy Experiments: DAPI/PI

For fluorescence microscopy experiments, two nucleic acid stains were used: DAPI (4′,6-diamidino-2-phenylindole dihydrochloride; Sigma-Aldrich, Milan, Italy) and propidium iodide (PI; Sigma-Aldrich, Milan, Italy). Briefly, 100 µL of bacterial cultures of *E. coli* and *S. aureus* (model Gram-negative and Gram-positive strains, respectively) were incubated at 37 °C for 4 h under shaking conditions, in the presence or absence of **As** and its individual components at MIC. Following incubation, 10 µL of each bacterial suspension was stained with a solution containing DAPI (1 µg/mL) and PI (20 µg/mL). Samples were incubated in the dark prior to analysis and then immediately observed under a fluorescence microscope (Olympus BX51, Olympus, Tokyo, Japan) equipped with a DAPI filter set (excitation/emission: 358/461 nm).

Image acquisition was performed using a digital camera (Olympus DP70) with a standardized exposure time of 500 ms for dual DAPI/PI staining. All images were acquired and processed according to the method described by Castagliuolo et al. [36].

### 2.9. Biofilm Activity

The antibiofilm activity of **As** and its principal components was evaluated against *M. smegmatis* biofilms using the colorimetric assay with crystal violet. Untreated microbial cells served as negative controls, while cultures treated with the antibiotic kanamycin were used as positive controls. A 24-well plate was prepared and incubated at 37 °C for 72 h in the presence of **As** and its individual components at concentrations ranging from 0 to 5 mg/mL to evaluate its ability to inhibit biofilm formation. Each well contained a final volume of 1 mL; the bacterial suspension was inoculated to reach an initial optical density (OD_600_) of 0.1, and variable volumes of **As** or individual compounds were added according to the tested concentration.

After incubation, biofilm biomass was quantified by crystal violet staining, and absorbance was measured at 570 nm using a Multiskan microplate reader (Thermo Electron Corporation, Waltham, MA, USA) [37]. To account for variations in planktonic growth, the results were normalized by calculating the ratio between the optical density at 570 nm (biofilm biomass) and the OD at 600 nm (planktonic cell density). The percentage of biofilm inhibition was determined by comparing the normalized optical density (OD) values of the treated samples with those of the untreated control samples.

### 2.10. DPPH Radical Scavenging Assay

The DPPH (2,2-diphenyl-1-picrylhydrazyl) radical scavenging activity was evaluated according to a previously described method, with minor modifications [38]. Different concentrations of **As** and its main constituents (2.5–10 mg/mL) were prepared in 100% methanol. For the assay, 100 µL of a freshly prepared DPPH solution (0.1 mM) and variable volumes of the tested samples (depending on the required concentration) were added to each tube. The final volume was consistently brought to 1 mL using 100% methanol, ensuring that the initial absorbance of the DPPH solution did not exceed 1.0 OD at 517 nm. The reaction mixtures were incubated at room temperature for 30 min in the dark, and absorbance was measured at 517 nm using a UV–Vis spectrophotometer.

The DPPH radical scavenging activity was calculated as follows:

DPPH scavenging activity (%) = 100 × (1 − AS/AC)

where AS is the absorbance of the sample, and AC is the absorbance of the control (DPPH solution without sample).

### 2.11. ABTS Radical Cation Scavenging Assay

The ABTS radical cation scavenging activity was determined according to a previously reported method [36]. Briefly, the ABTS stock solution was prepared by mixing 7 mM ABTS with 2.45 mM potassium persulfate and allowing the mixture to react in the dark at room temperature for 16 h. The resulting solution was diluted with phosphate-buffered saline (PBS) to obtain an absorbance of 0.72 ± 0.02 at 734 nm. For the test, varying volumes of **As** and of its individual components were used to achieve different concentrations (2.5–10 mg/mL), which were added to the 900 µL ABTS solution to reach the final volume of 1 mL, depending on the specific concentration tested, and were incubated in the dark at room temperature for 10 min. Absorbance was measured at 734 nm using a UV–Vis spectrophotometer (PharmaSpec, Shimadzu, Duisburg, Germany).

The ABTS radical scavenging activity was calculated as follows:

ABTS scavenging activity (%) = 100 × (1 − AS/AC)

where AC is the absorbance of the control (ABTS solution), and AS is the absorbance of the sample.

### 2.12. Hydrogen Peroxide Scavenging Assay

The hydrogen peroxide scavenging activity of **As** and its major constituents was evaluated using the method described by Beers and Sizer [39], with modifications by Petruk et al. [40]. For the assay, variable volumes of the tested samples (depending on the required concentration, 2.5–10 mg/mL) were mixed with a fresh H_2_O_2_ solution (43 mM H_2_O_2_ in 0.1 M potassium phosphate buffer, pH 7.0), and the mixture was brought to a final volume of 500 µL. The reaction mixtures were incubated at 20 °C for 30 min.

After incubation, the samples were centrifuged at 13,000× *g* for 1 min, and the supernatant was analyzed to determine the residual hydrogen peroxide by measuring the absorbance at 240 nm.

The scavenging activity was calculated as follows:

H_2_O_2_ scavenging activity (%) = 100 × (1 − AS/AC)

where AC is the absorbance of the control (H_2_O_2_ solution without sample), and AS is the absorbance of the tested sample.

### 2.13. Lipid Peroxidation Inhibition Assay

Lipid peroxidation inhibition was evaluated using the TBARS assay following previously reported methods [41,42].

Egg yolk homogenate (1:25, *v*/*v*, in PBS, pH 7.4) was used as lipid substrate. Reaction mixtures (460 µL final volume) contained 100 µL of homogenate, 10 µL of the test sample (**As**, its individual components, and ascorbic acid as a positive control), 50 µL of FeSO_4_ (25 mM), and PBS. The blank was prepared without FeSO_4_.

After incubation (37 °C, 15 min), the reaction was stopped with TCA (15% *w*/*v*), followed by the addition of acetic acid (20%, pH 3.5) and TBA (0.8% *w*/*v* in 1.1% SDS). Samples were heated (95 °C, 60 min), extracted with 1-butanol, and centrifuged. Absorbance of the organic phase was measured at 532 nm.

Samples were tested at concentrations ranging from 2.5 to 10 mg/mL, and results were expressed as inhibition of lipid peroxidation relative to the oxidized control.

### 2.14. Eukaryotic Cell Culture

Caco-2 cells (human colorectal adenocarcinoma cell line), purchased from CLS Cell Lines Service (Eppelheim, Germany), were maintained in Dulbecco’s Modified Eagle Medium (DMEM) (61965-026, GIBCO, Grand Island, NY, USA) and supplemented with 10% FBS (Euroclone, ECS500L, Pero, Italy), 1% Penicillin-Streptomycin (ECB3001D, Euroclone, Pero, Italy), and 4 mM L-Glutamine (ECB3000D, Euroclone, Pero, Italy). Cells grew at 37 °C in a humid atmosphere of 5% (*v*/*v*) CO_2_.

### 2.15. Cell Viability Assay

Caco-2 cells were seeded in 96-well plates overnight. The following morning, cells were treated with **As** and its individual components for 24 h at the concentrations corresponding to the highest MIC values, followed by the standard using the 3-[4,5-dimethylthiazol-2-yl]-2,5-diphenyl tetrazolium bromide (MTT) assay (M6494, Invitrogen, Eugene, OR, USA). Cells were incubated for 4 h at 37 °C in the dark with a culture medium containing 500 µg/mL of MTT solution. Subsequently, the medium was replaced with DMSO and incubated for another 10 min at 37 °C. Finally, the absorbance at 570 nm was determined using a plate reader (Synergy™ H4, Agilent BioTek, Santa Clara, CA, USA).

### 2.16. Oxidative Stress Measurement

Caco-2 cells were seeded in 96-well plates overnight. The following morning, the culture media were changed with fresh DMEM without phenol red (ECB7504L, Euroclone, Pero, Italy) supplemented with 10% FBS (Euroclone, ECS500L, Pero, Italy) and with **As**, camphor, 4-terpineol, and eucalyptol for 24 h, at MIC values. Additionally, 45 min before H_2_O_2_ treatment, 2′,7′-Dichlorofluorescin diacetate (Merk Life Science, Milano, Italy) was added to the culture media at a final concentration of 30 µM. After 45 min, the culture media were replaced with fresh DMEM without phenol red supplemented with 2% FBS.

Then, the cells were treated with 1 mM of H_2_O_2_ for 1.5 h. The fluorescence intensity (535 nm) was measured using a Synergy H4 Hybrid Microplate reader (Agilent, Santa Clara, CA, USA) [43].

### 2.17. In Silico Prediction of Biological Activity

To predict the potential pharmacological effects and molecular targets associated with the compounds identified in the **As**, the PASS (Prediction of Activity Spectra for Substances) online platform was employed [42,44].

For each compound, predicted activities are expressed as probability values: Pa (probability to be active) and Pi (probability to be inactive). In this study, only those predicted activities with Pa ≥ 0.7 were considered significant and retained for further analysis, as this threshold indicates a high likelihood of biological relevance.

### 2.18. Statistical Analysis

Statistical analysis was performed by one-way ANOVA with Tukey’s multiple comparisons test. All experiments were performed in three biological replicates. (ns: not significant; * *p* ≤ 0.05, ** *p* ≤ 0.01, *** *p* ≤ 0.001, **** *p* ≤ 0.0001).

## 3. Results and Discussion

### 3.1. Chemical Composition by Gas Chromatography and Mass Spectrometry (GC-MS) and 1D, 2D-NMR Analyses

The composition of **As** was analyzed by GC-MS. Nineteen compounds, divided into four classes, were identified (Table 1) and classified according to Linear Retention Indices (LRIs). A total of 97.3% of the composition was successfully identified by comparison of the indices and mass spectral data. In addition, to further confirm the composition, major compounds, camphor, eucalyptol, and 4-terpineol, were confirmed by injection of authentic standards. Only 2.7% of the total compounds were unidentified. In terms of compound classes, oxygenated monoterpenes (72.3%) dominate, by far, and then **As**, with camphor as the most abundant compound (31.3%), followed by 4-terpineol (11.3%), eucalyptol (10.6%), and *trans*-jasmone (6.4%).

Among the monoterpene hydrocarbons, camphene (5.3%) is present in major quantity, although similar amounts of *γ*-terpinene (4.0%), *α*-pinene (3.9%), *β*-pinene (3.1%), *α*-terpinene (3.1%), and *p*-cymene (3.1%) were also identified. On the other hand, sesquiterpenes, both hydrocarbons and oxygenated, occur in very moderate amounts: 0.4% and 1.2%, respectively.

**Figure 2:**
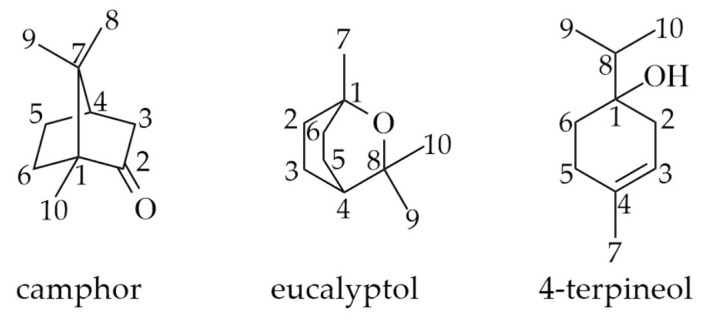
Molecular structures of camphor, eucalyptol, and 4-terpineol.

The compositional confirmation of the major compounds was also carried out using NMR spectroscopic techniques, in particular ^13^C-NMR and HMBC (Heteronuclear Multiple Bond Correlation). These three compounds, belonging to the class of oxygenated monoterpenes, have strictly related structures (Figure 2), although with different functional groups.

**Figure 3:**
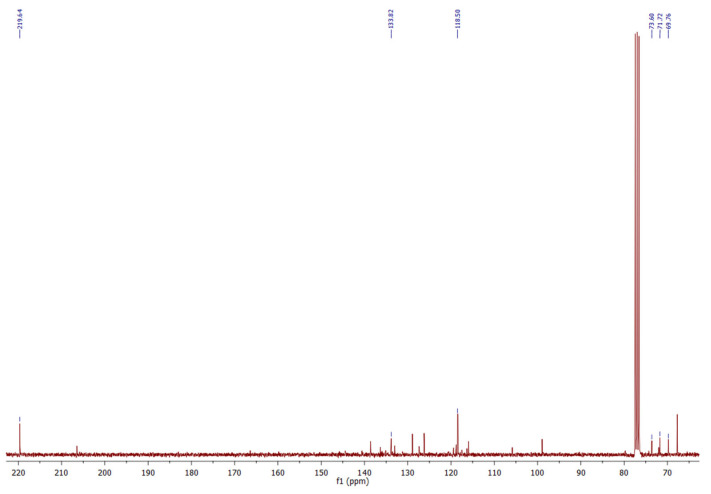
^13^C-NMR spectrum of *Achillea setacea* essential oil.

**Figure 4:**
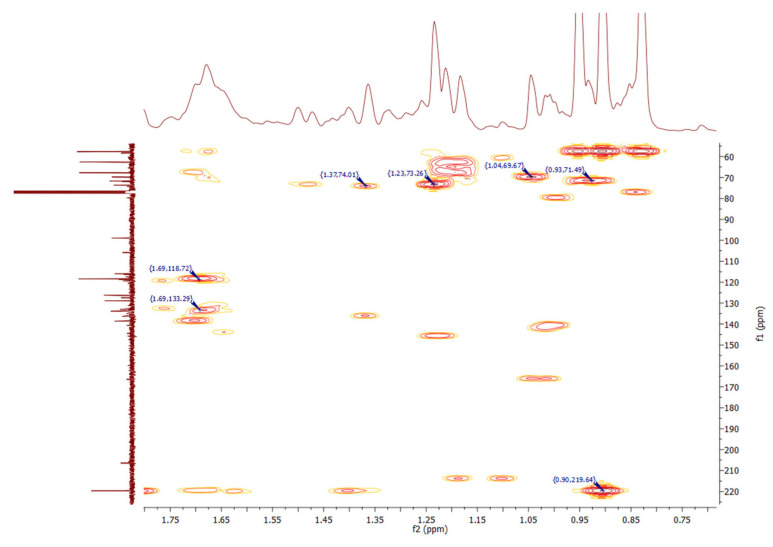
HMBC spectrum of *Achillea setacea* essential oil (**As**).

Indeed, camphor presented with a characteristic ketone peak in the carbon spectrum (Figure 3) at 219.64 ppm (C-2), which is confirmed by a clear correlation in the HMBC spectrum (Figure 4) with the C-10 methyl protons at 0.90 ppm. In turn, eucalyptol presents two quaternary carbons (C-1 and C-8) (Figure 3) close to oxygen, which resonate at 73.60 and 69.76 ppm, respectively. These two signals presented clear ^2^*J_CH_* correlations with the methyl protons H-7 (1.04 ppm) and H-9 and H-10 (1.23 ppm) (Figure 4).

Finally, 4-terpineol was structurally confirmed by the clear presence of two alkenyl carbons at 133.82 and 118.50 ppm (Figure 3), and both showed two correlation spots with methyl protons (C-7) at 1.69 ppm, through ^2^*J* and ^3^*J* correlations, respectively (Figure 4). C-1, in turn, presented with a clear peak at 71.72 ppm, accounting for an oxygenated carbon (Figure 3), and showed a strong correlation spot with the two methyls C-9 and C-10 (0.93 ppm) (Figure 4).

The chemical shifts in camphor, eucalyptol, and 4-terpineol were also confirmed by literature data [45,46,47].

Several papers have been published on the chemical compositions of the essential oil of *A. setacea* collected in some countries (Table 2), but as stated before, none concern the North Macedonian accession. Camphor, the main constituent of **As**, was detected in large amounts (>10%) in the leaves’ EO of the plant collected in Romania [23] and in the flowers’ EO of four Turkish accessions [21], whereas it was totally absent in the aerial parts of EOs of plants collected in Hungary, Iran, and Italy [20,22,24]. 4-Terpineol occurred only, and in moderate quantity, in three Turkish accessions [21,26], whereas eucalyptol was the main metabolite in almost all the Turkish accessions [21,26] in plants collected in Romania [23], and China [28]. It must be pointed out that the oils from Iran [24] and Italy [22] showed very different profiles with respect to **As**; in fact, they were extremely rich in sesquiterpenes, whereas metabolites were practically absent in **As**.

### 3.2. Antimicrobial Properties

The antimicrobial activity of **As** was evaluated by determining the minimum inhibitory concentration (MIC) against all the selected strains. In addition to the model organisms *E. coli* and *S. aureus*, the microbial panel included *B. cereus*, *S. epidermidis*, *P. aeruginosa*, and the fungal strain *C. albicans*. This resulted in a comprehensive antimicrobial profile of **As** and its main constituents, camphor, eucalyptol, and 4-terpineol, against representative microorganisms associated with skin, gastrointestinal, and mucosal infections. In particular, **As** demonstrated broad-spectrum antimicrobial activity, with MIC values ranging from 5 to 10 mg/mL, as shown in Table 3.

Interestingly, an evaluation of the individual main components revealed distinct biological activity profiles against the tested microorganisms. Eucalyptol, at 10.6%, showed the greatest antimicrobial potency among the main compounds, with MIC values of 8 mg/mL against *E. coli* and 16 mg/mL against *S. aureus*, as reported in Table 4.

In contrast, camphor (31.3%) and 4-terpineol (11.3%) showed slightly lower and more variable inhibitory effects despite being present in higher % concentrations. Specifically, camphor showed MIC values of 13 mg/mL and 16 mg/mL against *E. coli* and *S. aureus*, respectively. Similarly, 4-terpineol showed MIC values of 12 mg/mL against *E. coli* and 16 mg/mL against *S. aureus*. These results suggest that, although all three major metabolites contribute to the overall biological activity of **As**, eucalyptol plays a primary role in the observed antibacterial efficacy, particularly against *E. coli*. Furthermore, the greater activity of **As** compared to some individual components, treated in the concentrations (%) in which they are present in the EO, may suggest that the activity of the whole EO cannot be attributed to a single major constituent alone, a well-known characteristic of complex phytochemical mixtures [48].

However, the biological relevance of the observed MIC values should be interpreted with caution. Although **As** exhibited antimicrobial activity against all tested microorganisms, concentrations required to achieve complete growth inhibition were relatively high compared with those reported for conventional antimicrobial agents (positive control). Furthermore, the major constituents were evaluated at levels corresponding to their relative abundance within the essential oil rather than as pure compounds at a full concentration.

Furthermore, the antimicrobial activity observed for the **As** should also be interpreted in light of the pronounced chemical variability previously reported for this species. Different geographical populations have been shown to possess markedly distinct chemotypes, characterized by varying proportions of eucalyptol, camphor, borneol, and other oxygenated terpenes [21,23,26,27]. For example, Turkish populations were reported to contain high levels of 1,8-cineole (34.3–48.5%), whereas a camphor-rich chemotype (30.2%) was identified in samples collected from Karamenderes et al. [21]. Similarly, a Romanian chemotype was characterized by borneol (32.97%), eucalyptol (14.94%), and camphor (10.13%) as major constituents [23]. Previous studies reported antimicrobial activity against selected bacterial and fungal strains, with MIC values generally lower than those observed in the present work [23,26]. Such differences may be related to variations in chemical composition among chemotypes, as well as differences in the tested microorganisms and experimental conditions. Therefore, the antimicrobial profile of **As** appears to be strongly influenced by its phytochemical composition, highlighting the importance of chemotype-specific biological evaluation.

### 3.3. Mechanism of Action: Bacteriolytic Activity and Membrane Alterations

**Figure 5:**
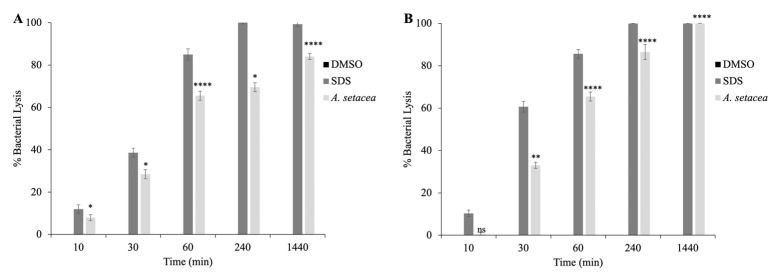
Bacterial lysis activity of **As**. Panel (**A**) against *E. coli* and panel (**B**) against *S. aureus*. **As** was tested at their respective MICs. DMSO 50% served as a negative control, while SDS 1% served as a positive control. Bacterial lysis was expressed as a percentage and evaluated over time (from 10 min to 24 h). Data represent the means of three independent experiments (*n* = 3). Statistical analysis was performed by one-way ANOVA with Tukey’s multiple comparisons test. ns: not significant; * *p* ≤ 0.05; ** *p* ≤ 0.01; **** *p* ≤ 0.0001.

To clarify the mechanisms underlying the antimicrobial activity of **As** and its main constituents, its potential ability to induce bacterial lysis was studied using an in vitro lysis assay, while alterations in membrane integrity were assessed by fluorescence microscopy. In particular, the lytic effect of **As** (Figure 5) showed that bacterial lysis occurred in both model strains, *E. coli* and *S. aureus*, already at short incubation times (30 min). However, interestingly, while in *S. aureus* the lytic effect became more pronounced with longer incubation times, in *E. coli*, it seemed to stabilize over time. This difference suggests a more immediate interaction of **As** with the cell envelope of Gram-positive bacteria, which is more accessible due to the absence of an external membrane barrier.

**Figure 6:**
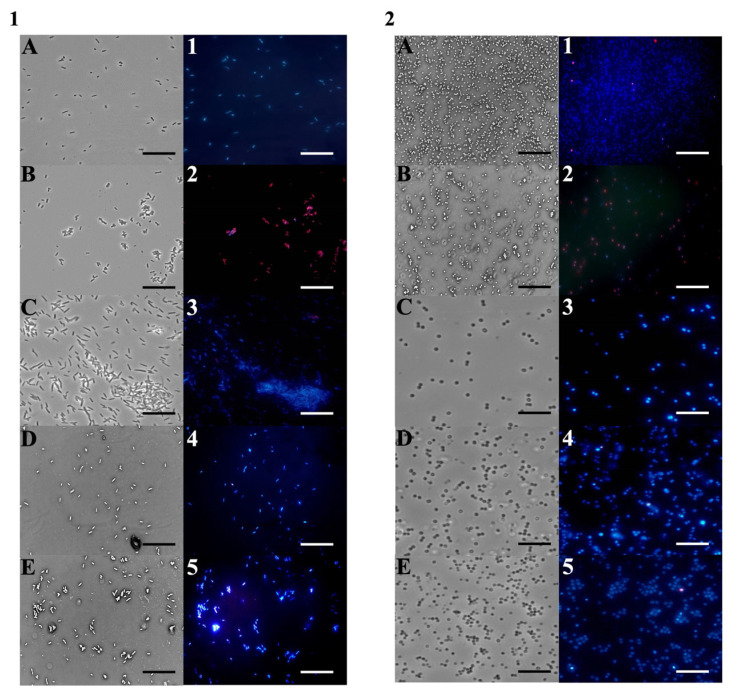
Fluorescence microscopy of *E. coli* cells (panel **1**) and *S. aureus* cells (panel **2**) after treatment with **As** Panel 1-2 (**B-2**), camphor Panel 1-2 (**C-3**), 4-terpineol Panel 1-2 (**D-4**) and eucalyptol Panel 1-2 (**E-5**), stained with DAPI and propidium iodide. Untreated control cells are shown in Panel 1-2 (**A-1**). Scale bars: 5 µm.

To further validate these observations and assess membrane damage, fluorescence microscopy analyses were performed using dual DAPI/propidium iodide (PI) staining. This approach enabled a more accurate evaluation of membrane integrity in both Gram-negative and Gram-positive bacterial cells, providing deeper insight into the antimicrobial mechanism of action. Specifically, the DAPI/PI dual staining assay was conducted on *E. coli* and *S. aureus*. As shown in Figure 6, untreated *E. coli* cells (Panel 1, A-1) exhibited a predominant blue fluorescence, indicative of intact membranes. In contrast, exposure to the **As** (Panel 1, B-2) resulted in a marked shift, with the appearance of intense red fluorescence, confirming the disruption of the bacterial membrane. This observation indicates increased membrane permeability, leading to cell death.

The individual constituents induced markedly different responses. Treatment with camphor (Panel 1, C-3) preserved a predominantly blue fluorescence. Similarly, 4-terpineol and eucalyptol (Panel 1, D-4 and E-5) showed mainly blue fluorescence, comparable to the control group. A comparable trend was observed in *S. aureus* (Panel 2). Control cells displayed typical blue fluorescence (Panel 2, A-1), whereas treatment with the complete **As** led to widespread red fluorescence (Panel 2, B-2), indicating effective membrane disruption also in Gram-positive bacteria. This result demonstrates that the essential oil can compromise bacterial membranes regardless of structural differences in the cell envelope, including the thick peptidoglycan layer characteristic of Gram-positive organisms. In contrast, the individual compounds, camphor (Panel 2, C-3), 4-terpineol (Panel 2, D-4), and eucalyptol (Panel 2, E-5), again produced predominantly blue fluorescence, confirming their limited ability to induce membrane permeabilization when tested separately. Overall, these findings moderately suggest that the antimicrobial activity of **As** is not attributable to a single dominant compound, but rather to several interactions among its constituents.

Therefore, the relatively high proportion of minor compounds, such as monoterpene hydrocarbons and oxygenated sesquiterpenes, may further contribute to biological activity through several interactions [49,50]. Such interactions may enhance the EO’s ability to penetrate and destabilize the lipid bilayer, causing irreversible membrane damage, explaining the red fluorescence observed in samples treated with **As** compared to those treated with the major components, which instead produced blue fluorescence. This effect is a well-known characteristic of EOs, where the combined action of multiple components, including both major and minor compounds, can lead to greater bioactivity than individual constituents [51,52]. Furthermore, minor components, even when present in low concentrations, can modulate membrane fluidity, facilitate the absorption of active compounds, or interfere with cellular homeostasis, thus amplifying the overall antimicrobial effect [50]. Therefore, these results support the hypothesis that *A. setacea* EO acts as a phytocomplex through a multifactorial mechanism involving both physical disruption of the bacterial membrane and possible interference with intracellular targets.

### 3.4. Antibiofilm Activity

**Figure 7:**
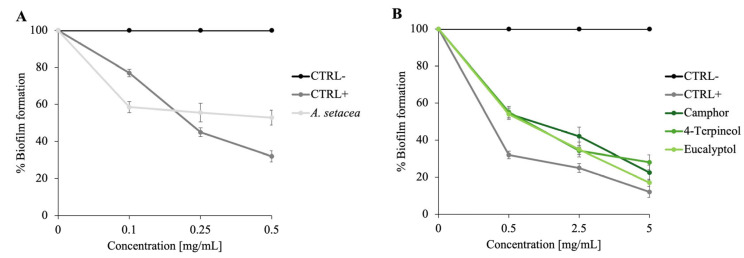
Inhibition of biofilm formation. Panel (**A**) shows the activity of **As**, while Panel (**B**) reports the effects of its individual components against *M. smegmatis* biofilms. Biofilm biomass was quantified following treatment, with concentrations ranging from 0.1 to 5 mg/mL. Untreated cells were used as a positive control (CTRL+), whereas cells treated with kanamycin served as a negative control (CTRL−). Data are presented as the mean of three independent experiments.

Subsequently, the antibiofilm potential of **As** and its individual components was studied. *M. smegmatis* was selected as a robust model organism for biofilm dynamics due to its ability to form complex, organized microbial structures, mirroring the persistence mechanisms observed in skin- and gut-associated pathogens [53,54]. To evaluate the specific antibiofilm activity of the EO, different concentrations were employed. In particular, as shown in Figure 7, concentrations between 0 and 5 mg/mL were tested to evaluate the ability of **As** (Figure 7A) and the individual components (Figure 7B) to interfere with the early stages of biofilm formation. The results show that the entire EO exerts a potent, dose-dependent inhibitory effect on biofilm development.

Specifically, even at a minimum concentration of 0.1 mg/mL, total oil was able to reduce biomass formation by approximately 40%, reaching an inhibition of over 60% at 0.5 mg/mL. These data highlight how the phytocomplex effectively interferes with the assembly of the matrix at extremely low doses. Analysis of the individual constituents revealed a different activity profile and lower potency than the total oil. Although eucalyptol, 4-terpineol, and camphor showed a progressive reduction in biofilm, they required concentrations of 0.5 mg/mL to achieve an inhibition of approximately 45%, a lower result than that obtained with the total EO. Only by increasing the concentration of the individual components to 5 mg/mL was a reduction in biomass of between 70% and 80% observed. These findings indicate that the biological activity of the complete phytocomplex differs from that of its isolated constituents and highlight the complexity of the EO composition. The coordinated interaction of the various molecules within the phytocomplex appears to more efficiently target the early stages of adhesion and the synthesis of the matrix, giving the EO a greater ability to interfere with the organized growth of mycobacteria than its individual isolated components.

### 3.5. Antioxidant Activity of **As** and Single Components

It is well documented in the literature that EOs, in addition to their well-known antimicrobial properties, can also exhibit antioxidant potential [36]. Considering this evidence, the antioxidant activity of **As** and its main constituents was investigated. The radical scavenging activity was evaluated using three complementary spectrophotometric assays: the DPPH (2,2-diphenyl-1-picrylhydrazyl) assay, hydrogen peroxide scavenging assay, and ABTS (2,2′-azino-bis(3-ethylbenzothiazoline-6-sulfonic acid)) assay. These widely used methods assess the ability of antioxidants to neutralize different reactive species through electron or hydrogen donation mechanisms [55].

**Figure 8:**
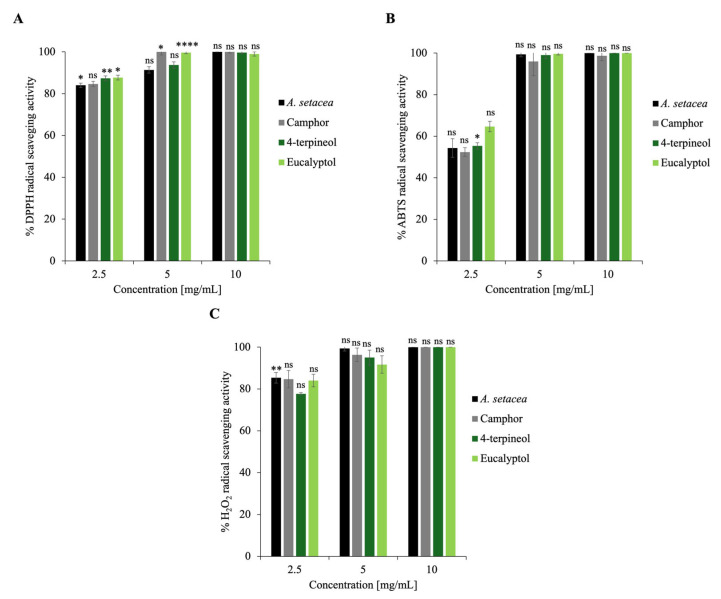
Antioxidant activities of **As**, camphor, 4-terpineol, and eucalyptol: (**A**) DPPH assay; (**B**) ABTS assay, and (**C**) H_2_O_2_ scavenging assay. All samples were tested at concentrations ranging from 2.5 to 10 mg/mL. Data represent the means of three independent experiments (*n* = 3). Statistical analysis was performed by one-way ANOVA with Tukey’s multiple comparisons test. ns: not significant; * *p* ≤ 0.05; ** *p* ≤ 0.01; **** *p* ≤ 0.0001.

As shown in Figure 8, **As** exhibited a moderate dose-dependent antioxidant activity across all assays. In particular, in the DPPH and hydrogen peroxide (H_2_O_2_) scavenging assays (Figure 8A,C), **As** demonstrated efficacy already at 2.5 mg/mL, exceeding 80% radical scavenging activity and approaching nearly complete inhibition (≈100%) at 5 mg/mL, especially in the H_2_O_2_ assay. A similar trend was observed in the ABTS assay (Figure 8B), where antioxidant activity increased consistently with concentration, reaching high levels at intermediate doses and maximal effects at the highest concentration tested.

Comparison with the individual components, camphor, 4-terpineol, and eucalyptol, showed that all compounds exhibit a dose-dependent increase in antioxidant activity across the three assays. While the whole **As** consistently displays high scavenging activity, particularly at higher concentrations, some individual components reach comparable levels under certain conditions. In particular, 4-terpineol showed antioxidant activity close to that of **As** at higher concentrations, whereas eucalyptol exhibits strong activity across all assays, especially in ABTS and H_2_O_2_. Camphor, on the other hand, generally shows slightly lower activity, although it still follows a clear dose-dependent trend. These observations agree with the IC_50_ values reported in Table 5.

Indeed, eucalyptol 10.6% displays the lowest IC_50_ in the ABTS assay (1.5 ± 0.5 mg/mL), indicating higher radical scavenging efficiency compared to the essential oil (**As**, 2.5 ± 0.2 mg/mL) and the other components. In the DPPH assay, all samples show comparable IC_50_ values (1.2–1.7 mg/mL), while in the H_2_O_2_ assay, **As**, camphor, and eucalyptol exhibit similar IC_50_ values (~1.5 mg/mL), with 4-terpineol showing slightly higher values (2.0 ± 0.3 mg/mL), suggesting lower efficiency in this test. As expected, ascorbic acid presents markedly lower IC_50_ values in all assays, confirming its much higher antioxidant potency compared to both **As** and its individual constituents. Overall, these results indicate that the antioxidant activity of **As** reflects the combined contribution of multiple components, with eucalyptol and 4-terpineol playing an important role, rather than being driven by a single dominant compound.

This finding suggests that the antioxidant properties of EO cannot be attributed solely to a single major component. Indeed, it is widely recognized that the biological activity of EOs reflects the contribution of both major and minor constituents [49,56,57]. However, since the specific interactions among the components were not investigated in the present study, no conclusions can be drawn regarding possible synergistic or additive effects. Overall, these findings demonstrate that **As** possesses measurable antioxidant activity in vitro and thus support further studies aimed at clarifying the contribution of individual constituents and the biological relevance of the observed effects.

**Figure 9:**
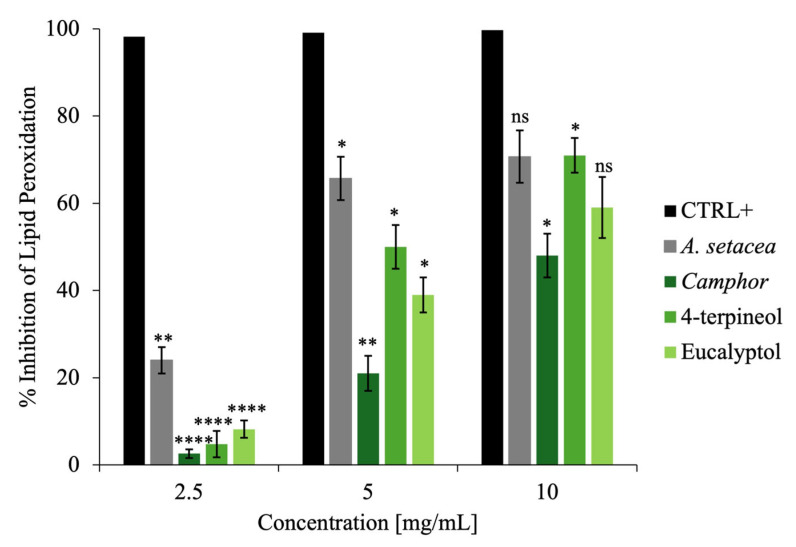
Antioxidant activity of **As**, camphor, 4-terpineol, and eucalyptol evaluated by the TBARS assay in an egg yolk lipid peroxidation system. All samples were tested at concentrations ranging from 2.5 to 10 mg/mL. Data represent the means of three independent experiments (*n* = 3). Statistical analysis was performed by one-way ANOVA with Tukey’s multiple comparisons test, analyzing each sample against the Ascorbic acid treatment (CTRL+). ns: not significant; * *p* ≤ 0.05; ** *p* ≤ 0.01; **** *p* ≤ 0.0001.

While the DPPH, ABTS, and H_2_O_2_ assays evaluate the radical scavenging capacity of the samples in chemical systems, they do not fully reflect antioxidant activity in complex lipid-rich environments. In contrast, the TBARS (thiobarbituric acid reactive substances) assay, performed using egg yolk as a lipid substrate, provides a more biologically relevant model by assessing the ability of the samples to inhibit lipid peroxidation processes. As shown in Figure 9, **As** exhibited a clear dose-dependent inhibition of lipid peroxidation, showing the highest activity among all tested samples at each concentration.

The individual components also displayed protective effects against lipid oxidation, although with different efficiencies. Camphor showed moderate inhibitory activity, whereas 4-terpineol and eucalyptol exhibited lower activity with respect to **As** at 2.5 and 5 mg/mL, but a marked increase at 10 mg/mL. In particular, 4-terpineol demonstrated slightly higher inhibition than eucalyptol at the highest tested concentration. Compared with the DPPH, ABTS, and H_2_O_2_ assays, these differences likely reflect the greater complexity of antioxidant mechanisms involved in lipid systems, where antioxidant compounds may act not only through direct radical scavenging but also by interacting with lipid substrates and interrupting lipid peroxidation chain reactions. Overall, the TBARS results further confirm the antioxidant potential of **As** in a model that better mimics biological oxidative processes.

While the DPPH, ABTS, H_2_O_2_, and TBARS assays provide useful information on the antioxidant potential of **As**, these methods are based on simplified chemical or cell-free systems and do not fully reproduce the complexity of physiological conditions. Therefore, the observed activities should be considered preliminary evidence of antioxidant in vitro potential.

Furthermore, the considerable phytochemical variability reported for **As** can also be discussed. Previous investigations have demonstrated substantial differences in the relative abundance of oxygenated monoterpenes and sesquiterpenes among chemotypes from different geographical regions [21,23,27]. In particular, Romanian populations were found to be enriched in borneol and eucalyptol, whereas Turkish populations frequently exhibited eucalyptol-rich chemotypes [21,23]. Since compounds such as eucalyptol, borneol, camphor, and terpinen-4-ol have been associated with antioxidant properties, differences in their relative abundance may contribute to the variability in antioxidant activity reported among studies. Moreover, investigations performed on different *Achillea* species have generally described low to moderate antioxidant activity of EOs in chemical assays, supporting the view that antioxidant performance is highly dependent on chemical composition and experimental conditions [27].

### 3.6. Cytotoxic Activity

**Figure 10:**
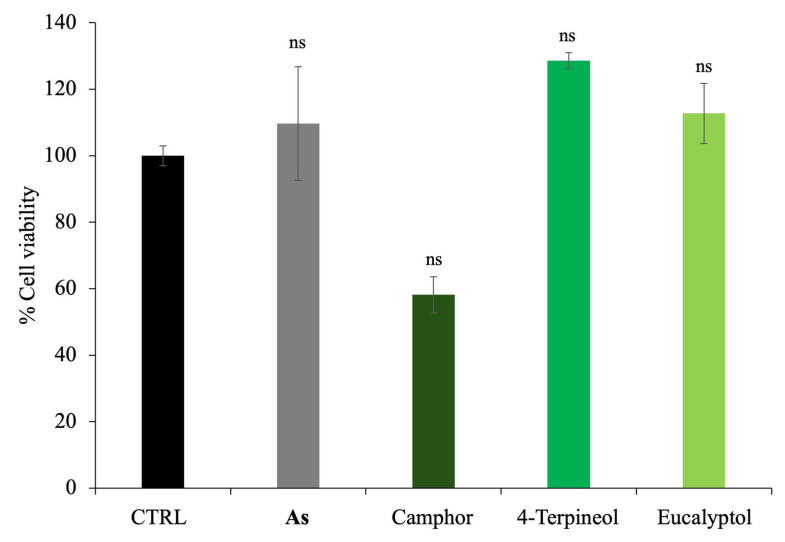
Effects of **As**, camphor, 4-terpineol, and eucalyptol on Caco-2 cell viability evaluated by the MTT assay. All compounds were tested at concentrations corresponding to the highest MIC values previously determined in antimicrobial assays. Cell viability was expressed as a percentage relative to DMSO 50% control cells (CTRL). Data represent the means of three independent experiments (*n* = 3). Statistical analysis was performed by one-way ANOVA with Tukey’s multiple comparisons test, analyzing each sample against DMSO treatment (CTRL). ns: not significant.

The effects of **As** and its main components on Caco-2 cell viability were evaluated using the MTT assay. All samples were tested at concentrations corresponding to the highest MIC values previously determined in antimicrobial assays. As shown in Figure 10, treatment with **As**, camphor, 4-terpineol, and eucalyptol generally maintained high levels of cell viability compared with DMSO control cells (CTRL).

However, some treatments induced a reduction in cell viability compared to the CTRL, such as camphor. Although **As,** eucalyptol, and 4-terpineol maintained relatively high levels of cell viability, camphor induced a reduction in Caco-2 viability under the tested conditions. Considering that camphor represents the major constituent of **As** (31.3%), this finding suggests that individual components may differ substantially in their cytotoxic profiles. Notably, the whole **As** sample exhibited a more favorable viability profile than camphor tested individually at the corresponding concentration. This observation highlights that the biological effects of the complete phytocomplex cannot be directly inferred from those of a single constituent. Therefore, while **As** showed limited cytotoxicity at the tested concentrations, this observation cannot be generalized to all its individual constituents, and further studies are needed to better characterize their safety profiles.

### 3.7. Antioxidant Activity of **As** on Eukaryotic Cells

**Figure 11:**
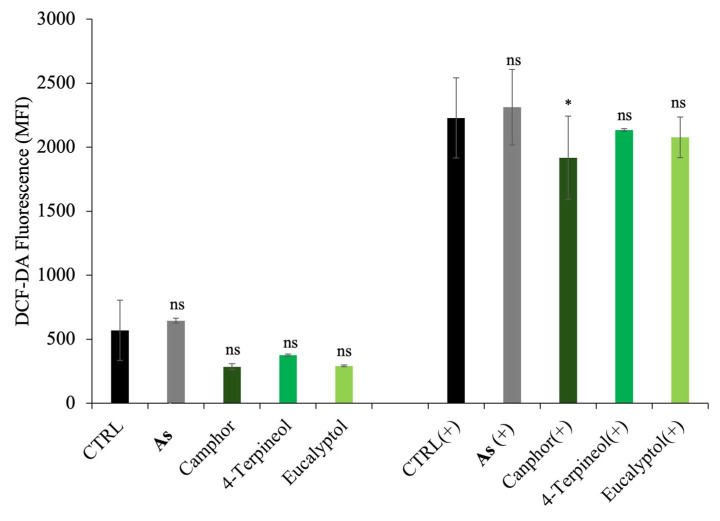
Intracellular antioxidant activity of **As**, camphor, 4-terpineol, and eucalyptol in Caco-2 cells evaluated by the DCF-DA fluorescence assay. All compounds were tested at the highest MIC values previously determined. (+) indicates cells treated with H_2_O_2_-induced oxidative stress. Data represent intracellular ROS levels expressed as mean fluorescence intensity (MFI) from three independent experiments. Data represent the means of three independent experiments (*n* = 3). Statistical analysis was performed by one-way ANOVA with Tukey’s multiple comparisons test, analyzing each sample against each relative DMSO treatment (CTRL: w/o H_2_O_2_; CTRL+: with H_2_O_2_). ns: not significant; * *p* ≤ 0.05.

The antioxidant activity of **As** and its main components was further evaluated in Caco-2 cells by measuring intracellular reactive oxygen species (ROS) production through the DCF-DA fluorescence assay. **As** shown in Figure 11, under basal conditions, all tested samples exhibited low fluorescence values comparable to the control, indicating the absence of marked pro-oxidant effects. Among the tested compounds, camphor, 4-terpineol, and eucalyptol showed lower fluorescence levels compared to DMSO or ***As***-treated cells, suggesting a reduction in basal intracellular ROS levels.

Following oxidative stress induction with H_2_O_2_ (+), a marked increase in DCF-DA fluorescence was observed, confirming enhanced intracellular ROS production. Treatment with **As** in the presence of H_2_O_2_ did not result in a reduction in ROS levels compared with the control. Instead, 4-terpineol and eucalyptol showed moderate decreases in fluorescence intensity. Notably, camphor produced the strongest antioxidant effect among the tested compounds under oxidative conditions, reducing ROS generation compared with DMSO-treated cells. Overall, these findings indicate that **As** and its individual components exert protective antioxidant effects in Caco-2 cells, particularly under oxidative stress conditions induced by H_2_O_2_.

### 3.8. Prediction of Biological Activity

Using the PASS (Prediction of Activity Spectra for Substances) online tool, the three major constituents of **As**, camphor, 4-terpineol, and eucalyptol, were screened for their potential biological activities. Only activities with a probability (Pa) ≥ 0.7 were considered significant and are reported in Table 6.

Overall, the PASS analysis identified several predicted pharmacological activities that may be relevant for interpreting the biological properties of the major constituents of **As**. However, these predictions should be regarded as hypothesis-generating tools and not as experimental evidence of biological activity or a mechanism of action. The predicted activities provide possible directions for future investigations aimed at validating the molecular targets and pathways involved.

Camphor exhibited predicted activities mainly related to skin-related conditions and membrane-associated mechanisms, including antiseborrheic and antieczema tic effects, as well as membrane permeability modulation. These properties are in agreement with its traditional topical use [58,59]. Furthermore, the predicted stimulation of the NF-E2-related factor 2 (Nrf2) pathway suggests a possible involvement in cellular antioxidant defense mechanisms, consistent with the radical scavenging activity observed in the DPPH, ABTS, and H_2_O_2_ assays [60].

Similarly, 4-terpineol showed a strong association with dermatological and anti-inflammatory activities, including antieczema tic and antiseborrheic properties, along with the inhibition of matrix metalloproteinase-9 (MMP9) expression. The modulation of MMP9 is particularly relevant, as this enzyme plays a key role in extracellular matrix degradation and inflammatory processes [61]. In addition, predicted effects on membrane integrity and proton-transporting ATPases suggest a mechanism involving bacterial membrane destabilization, which may contribute to the observed antibiofilm and antimicrobial activity.

Eucalyptol displayed predicted activities mainly related to mucosal protection, cellular stress response, and redox balance, including mucomembranous protective effects and inhibition of hypoxia-inducible factor 1-alpha (HIF1A) expression. These findings are consistent with the well-known pharmacological profile of eucalyptol as a respiratory and anti-inflammatory agent [62]. Moreover, its predicted involvement in mitochondrial electron transport processes further supports its contribution to the antioxidant activity observed experimentally. Taken together, the PASS predictions suggest that the biological activity of **As** is not attributable to a single compound, but rather to the complementary and potentially synergistic actions of its major constituents. In particular, the convergence of membrane-targeting effects, antioxidant pathways, and dermatological activities provides a coherent mechanistic explanation for the antimicrobial, antibiofilm, and antioxidant properties observed in this study.

## 4. Conclusions

This study provides the first comprehensive phytochemical and biological characterization of the essential oil of wild *Achillea setacea* from North Macedonia. This study demonstrates the consistency and robustness of both spectrometric and spectroscopic approaches, such as GC-MS and NMR techniques. Qualitative GC-MS analysis confirmed the presence of three major compounds: camphor, 4-terpineol, and eucalyptol, which were subsequently structurally confirmed through 1D- and 2D-NMR analyses. The essential oil showed interesting antimicrobial activity against Gram-positive, Gram-negative, fungal, and mycobacterial strains, along with a marked ability to inhibit biofilm formation. Fluorescence microscopy and bacterial lysis tests suggest that membrane destabilization represents one of the oil’s primary mechanisms of action, and specifically, it consistently showed stronger activity than the main compounds tested. In antioxidant tests (DPPH, ABTS, and H_2_O_2_), the essential oil showed strong free radical scavenging activity, while the TBARS test further demonstrated its ability to inhibit lipid peroxidation. Importantly, intracellular ROS measurements in Caco-2 cells confirmed that the oil’s main constituents are also capable of maintaining oxidative stress levels comparable to those of the control under H_2_O_2_-induced conditions. Finally, the essential oil showed limited cytotoxicity against Caco-2 cells at concentrations corresponding to the highest antimicrobial MIC values, indicating acceptable biocompatibility. In silico PASS predictions further supported the experimental results, suggesting activities related to antioxidant defense, membrane modulation, antimicrobial action, and dermatological applications. Overall, although the promising data presented in this work represent a starting point requiring further in vivo and multi-regional investigations to validate these findings, this study establishes a solid scientific foundation for the potential exploitation of *A. setacea* essential oil in pharmaceutical and cosmetic formulations.

## Figures and Tables

**Table 1:** Chemical composition of *Achillea setacea* essential oil (**As**) collected in North Macedonia.

No.	Compounds ^a^	LRI ^b^	LRI ^c^	Area (%) ^d^
				As
1	*α*-Pinene	925	927	3.9
2	Camphene	941	943	5.3
3	*β*-Pinene	971	974	3.1
4	*α*-Terpinene	1012	1014	3.1
5	*p*-Cymene	1020	1019	3.1
6	Eucalyptol	1032	1031	10.6
7	*γ*-Terpinene	1054	1057	4.0
8	Terpinolene	1079	1083	1.0
9	Chrysanthenone	1096	1106	4.0
10	Camphor	1146	1145	31.3
11	Borneol	1176	1168	3.7
12	4-Terpineol	1182	1177	11.3
13	Carvone	1193	1195	0.7
14	*γ*-Terpineol	1198	1200	2.2
15	Verbenone	1238	1237	1.0
16	*cis*-Chrysanthenyl acetate	1253	1260	1.2
17	*trans*-Jasmone	1387	1391	6.4
18	*β*-Caryophyllene	1408	1413	0.4
19	Caryophyllene oxide	1572	1578	1.2
	Monoterpene Hydrocarbons			23.4
	Oxygenated Monoterpenes			72.3
	Sesquiterpene Hydrocarbos			0.4
	Oxygenated Sesquiterpenes			1.2
	Total			97.3

^a^ Components listed in order of elution on a DB-5 MS non-polar column; ^b^ experimental LRIs on a DB-5 MS non-polar column. ^c^ LRIs based on the literature (https://webbook.nist.gov/ (accessed on 26 March 2026); ^d^ calculated from the area of every single peak in the chromatogram.

**Table 2:** Main constituents (>3%) of the essential oils of *A. setacea* Waldst. & Kit reported in the literature.

Origin	Parts	Composition	Reference
China	ap	eucalyptol (7.3%), *γ*-selinene (6.2%), chamazulene (5.2%), camphor (4.3%), *δ*-3-carene (3.9%), *β*-pinene (3.4%)	[28]
Hungary	ap	chamazulene, *β*-pinene, eucalyptol, *β*-cubebene, farnesene, *β*-bisabolene, elemol	[20]
Iran	ap	nerolidol (20.0%), *α*-cubebene (14.0%), *δ*-cadinene (6.4%), germacrene D (5.5%), spathulenol (5.2%), lavandulyl acetate (5.1%), *β*-selinol (4.9%), *β*-selinene (4.4%), eucalyptol (4.1%), *α*-terpineol (3.1%)	[24]
Italy	ap	caryophyllene oxide (18.1%), *β*-eudesmol (12.9%), α-bisabolol (11.3%), viridiflorol (9.4%), germacrene D (5.6%), spathulenol (4.4%), bisabolene oxide (3.4%)	[22]
Romania	lv	borneol (33.0%), eucalyptol (14.9%), camphor (10.1%), artemisia ketone (4.7%), *α*-terpineol (3.2%), *γ*-eudesmol (3.2%)	[23]
Turkey	fl	eucalyptol (35.4%), borneol (11.7%), yomogi alcohol (11.0%), artemisia alcohol (7.4%), camphor (5.2%), bornyl acetate (5.0%), piperitone (4.5%), α-thujone (3.1%)	[21]
Turkey	fl	eucalyptol (48.5%), fragranol (17.2%), camphor (8.3%), borneol (4.6%), *α*-terpineol (3.5%), caryophyllene oxide (3.5%),	[21]
Turkey	fl	eucalyptol (34.3%), caryophyllene acetate (23.4%), -bisabolol oxide B (10.3%), camphor (7.4%), *α*-terpineol (4.5%), 4-terpinenol (3.9%)	[21]
Turkey	fl	eucalyptol (36.7%), camphor (16.2%), *α*-terpineol (9.7%), caryophyllene acetate (7.2%), camphene hydrate (4.6%), 4-terpinenol (4.0%), *β*-terpineol (3.9%)	[21]
Turkey	fl	camphor (30.2%), eucalyptol (12.7%), *epi*-α-bisabolol (7.6%), bornyl acetate (6.7%)	[21]
Turkey	fl	eucalyptol (38.2%), caryophyllene oxide (11.6%), camphor (11.5%), borneol (9.2%), α-thujone (7.2%), piperitone (3.1%),	[21]
Turkey	fl	eucalyptol (42.3%), camphor (15.6%), piperitone (11.4%), yomogi alcohol (5.1%)	[21]
Turkey	ap	α-bisabolone oxide A (27.0%), hexadecanoic acid (16.2%), α-bisabolol oxide B (5.7%), α-bisabolol (4.8%), camphor (4.1%), caryophyllene oxide (3.1%), eucalyptol (3.1%)	[25]
Turkey	ap	*β*-eudesmol (29.2%), α-bisabolone oxide A (26.4%), debromoallolaurinterol (14.9%), caryophyllene oxide (3.4%)	[27]
Turkey	ap	eucalyptol (18.5%), sabinene (10.8%), camphor (5.3%), bisabolone oxide (3.7%), 4-terpinenol (3.5%), *α*-terpineol (3.3%), borneol (3.1%)	[26]

ap = aerial parts; lv = leaves; fl = flowers.

**Table 3:** Minimum inhibitory concentrations (MIC) of **As** against selected strains. Colistin was used as a positive control against *P. aeruginosa* and *C. albicans*, while ampicillin was used against the other selected strains. Values represent the mean of three independent experiments and are expressed in mg/mL ± standard deviation (SD).

	As	Positive Control
Strains	MIC (mg/mL) ± SD	
*E. coli*	5 ± 0.08	0.002 ± 0.0005
*P. aeruginosa*	10 ± 0.09	0.05 ± 0.01
*S. aureus*	7 ± 0.05	0.002 ± 0.0001
*B. cereus*	6 ± 0.1	0.032 ± 0.002
*S. epidermidis*	6 ± 0.01	0.003 ± 0.0001
*C. albicans*	5 ± 0.02	0.064 ± 0.003
*M. smegmatis*	3 ± 0.05	0.2 ± 0.04

**Table 4:** Minimum inhibitory concentrations (MICs) of the main constituents (camphor, eucalyptol, and 4-terpineol). Values represent the mean of three independent experiments and are expressed in mg/mL ± standard deviation (SD).

MIC (mg/mL) ± SD			
	4-Terpineol (11.3%)	Camphor (31.3%)	Eucalyptol (10.6%)
*E. coli*	12 ± 0.07	13 ± 0.1	8 ± 0.09
*S. aureus*	16 ± 0.03	16 ± 0.05	16 ± 0.05
*M. smegmatis*	12 ± 0.8	10 ± 0.07	8 ± 0.03

**Table 5:** IC_50_ values (mg/mL ± SD) of **As**, camphor, 4-terpineol, eucalyptol, and ascorbic acid determined by DPPH, ABTS, and H_2_O_2_ radical scavenging assays. Data are expressed as mean ± standard deviation (SD) from three independent experiments. Lower IC_50_ values indicate higher antioxidant activity.

	IC_50_ [mg/mL] ± SD		
Sample	DPPH	ABTS	H_2_O_2_
*A. setacea*	1.7 ± 0.2	2.5 ± 0.2	1.5 ± 0.5
Camphor	1.5 ± 0.5	2.5 ± 0.4	1.5 ± 0.9
4-Terpineol	1.5 ± 0.7	2.5 ± 0.2	2 ± 0.3
Eucalyptol	1.2 ± 0.5	1.5 ± 0.5	1.5 ± 0.3
Ascorbic acid	0.03 ± 0.03	0.05 ± 0.06	0.04 ± 0.05

**Table 6:** Pharmacological activities of **As** major compounds by PASS Online: a computational assessment. Probable activity (Pa) and inactivity (Pi) thresholds: Only predictions with Pa > Pi were considered. Pa ≥ 0.7 represents a high probability of observable biological activity.

Compound	Pharmacological Effects Predictions	Pa	Pi
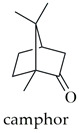	Antiseborrheic	0.877	0.006
	Antieczematic	0.746	0.031
	Membrane permeability inhibitor	0.747	0.022
	NF-E2-related factor 2 stimulant	0.706	0.002
	JAK2 expression inhibitor	0.743	0.013
	Mucomembranous protector	0.729	0.044
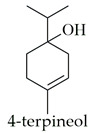	Antieczematic	0.838	0.011
	Antiseborrheic	0.796	0.020
	Membrane integrity agonist	0.737	0.048
	MMP9 expression inhibitor	0.715	0.006
	H+-exporting ATPase inhibitor	0.720	0.004
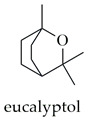	Mucomembranous protector	0.703	0.055
	HIF1A expression inhibitor	0.754	0.015
	Ubiquinol-cytochrome-c reductase inhibitor	0.858	0.015
	Carminative	0.790	0.004
	Alkenylglycerophosphocholine hydrolase inhibitor	0.808	0.017

## Data Availability

All data and materials are available upon request from the corresponding author.
